# Cystatin E/M suppresses legumain activity and invasion of human melanoma

**DOI:** 10.1186/1471-2407-10-17

**Published:** 2010-01-15

**Authors:** Jon J Briggs, Mads H Haugen, Harald T Johansen, Adam I Riker, Magnus Abrahamson, Øystein Fodstad, Gunhild M Mælandsmo, Rigmor Solberg

**Affiliations:** 1 Department of Tumor Biology, Institute for Cancer Research, Radiumhospitalet, Oslo University Hospital, Oslo, Norway; 2 Department of Pharmaceutical Biosciences, School of Pharmacy, University of Oslo, PO Box 1068 Blindern, N-0316 Oslo, Norway; 3 Ochsner Cancer Institute, Ochsner Medical Center, New Orleans, Louisiana, USA; 4 Department of Laboratory Medicine, Division of Clinical Chemistry & Pharmacology, Lund University, Sweden; 5 Radiumhospitalet Faculty Division, Medical Faculty, University of Oslo, Oslo, Norway

## Abstract

**Background:**

High activity of cysteine proteases such as legumain and the cathepsins have been shown to facilitate growth and invasion of a variety of tumor types. In breast cancer, several recent studies have indicated that loss of the cysteine protease inhibitor cystatin E/M leads to increased growth and metastasis. Although cystatin E/M is normally expressed in the skin, its role in cysteine protease regulation and progression of malignant melanoma has not been studied.

**Methods:**

A panel of various non-melanoma and melanoma cell lines was used. Cystatin E/M and C were analyzed in cell media by immunoblotting and ELISA. Legumain, cathepsin B and L were analyzed in cell lysates by immunoblotting and their enzymatic activities were analyzed by peptide substrates. Two melanoma cell lines lacking detectable secretion of cystatin E/M were transfected with a cystatin E/M expression plasmid (pCST6), and migration and invasiveness were studied by a Matrigel invasion assay.

**Results:**

Cystatin E/M was undetectable in media from all established melanoma cell lines examined, whereas strong immunobands were detected in two of five primary melanoma lines and in two of six lines derived from patients with metastatic disease. Among the four melanoma lines secreting cystatin E/M, the glycosylated form (17 kD) was predominant compared to the non-glycosylated form (14 kD). Legumain, cathepsin B and L were expressed and active in most of the cell lines, although at low levels in the melanomas expressing cystatin E/M. In the melanoma lines where cystatin E/M was secreted, cystatin C was generally absent or expressed at a very low level. When melanoma cells lacking secretion of cystatin E/M were transfected with pCST6, their intracellular legumain activity was significantly inhibited. In contrast, cathepsin B activity was not affected. Furthermore, invasion was suppressed in cystatin E/M over-expressing melanoma cell lines as measured by the transwell Matrigel assay.

**Conclusions:**

These results suggest that the level of cystatin E/M regulates legumain activity and hence the invasive potential of human melanoma cells.

## Background

Cystatins are endogenous inhibitors of cysteine proteases that form reversible, high-affinity complexes with their target proteases [1]. Loss of cystatin function results in deregulated activity of cysteine proteases and may lead to a variety of disorders, including chronic inflammatory reactions [2], faulty differentiation in epidermis [3], and tumor malignancy [4]. Cystatin E/M was originally identified as a cysteine protease inhibitor down-regulated in metastatic versus primary breast cancer cells [5,6], and subsequent studies have indicated that loss of cystatin E/M expression during the progression of breast cancer, glioma and lung cancer results from epigenetic silencing of the *CST6 *gene [7-11]. Restoration of cystatin E/M expression in breast cancer reduces growth, migration, and Matrigel invasion *in vitro *[12], as well as tumor growth and metastatic burden *in vivo *[13]. In glioma, expression of cystatin E/M inhibited cell motility and invasion *in vitro *[14]. Overall, these studies suggest a tumor suppressor function for this protease inhibitor.

Cystatin E/M exerts its inhibitory effects on two families of cysteine proteases, the papain-like cysteine proteases, including the cathepsins B, L, and V [15,16], and the asparaginyl endopeptidase (AEP) legumain, through two distinct binding sites [17]. The cathepsins are located in lysosomal compartments but may also be secreted to degrade extracellular matrix components [18]. Cathepsins B and L have been implicated in invasion and metastasis [19-21] and elevated levels have been observed in a variety of cancer types [22-24]. In melanoma, cathepsin B and L activities are increased relative to normal skin [25], and both the cysteine proteases conferred a metastatic phenotype [26,27]. Legumain is primarily a lysosomal enzyme, however, it has also been found on the cell surface and in membrane-associated vesicles concentrated along the invadopodia of tumor cells [28]. It is highly expressed in several cancer types including colon, prostate and breast cancer, and increased levels have been observed relative to adjacent non-neoplastic tissue in colorectal cancer [29]. Cells over-expressing legumain exhibit increased invasiveness both *in vitro *and *in vivo *[28], possibly attributed to the ability of legumain to activate proMMP-2 [28,30].

Our initial interest in cystatin E/M arose from microarray studies comparing the gene expression profiles of primary and metastatic melanoma [31], where expression of the cystatin E/M gene (*CST6*) was observed in normal skin and thin primary tumors, but not in thicker primaries or metastatic disease. This led us to hypothesize that cystatin E/M expression may be lost during the initial phases of melanoma tumor progression. To study the role of this protease inhibitor further, we utilized low-passage cell lines established from both primary and metastatic patient lesions. Cell lines were screened for secretion of cystatin E/M and C, as well as expressions of legumain, cathepsin B and L. We found that cystatin E/M, legumain, cathepsin B and L were expressed in several of these melanoma cell lines and that over-expression of cystatin E/M resulted in inhibition of legumain activity and decreased invasiveness.

## Methods

### Cell lines

Cell lines were established from freshly procured samples from patients with primary (MCC5, MCC13, MCC70, MCC80A) or metastatic (MCC11, MCC35, MCC52, MCC57, MCC69B, and MCC72) malignant melanoma at Moffitt Cancer Centre (MCC; Tampa, FL, USA) using standard *in vitro *cell culturing techniques [31]. The cell line DK928 was established from a patient with primary melanoma at the Mitchell Cancer Institute, University of South Alabama (Mobile, AL, USA). The following cell lines were obtained from patients with malignant melanoma treated at the Surgery Branch, Division of Clinical Sciences, National Cancer Institute (National Institutes of Health, Bethesda, MD, USA); 624.38 [32], 1495 [33], and 2139 (unpublished). The uveal melanoma cell line 92.1 was generously provided by Martine Jager (Leiden University Medical Centre, The Netherlands; [34]). The breast carcinoma cell line MA11 was established from bone marrow aspirate from a patient with lobular carcinoma [35], and the melanoma cell line WM239 was obtained from Meenhard Herlyn (The Wistar Institute, Philadelphia, PA, USA). A375 and SK-MEL-28 (both from malignant melanoma), DU145 (prostate carcinoma), HCT-116 (colorectal carcinoma), MCF7 (breast carcinoma) and MCF10A (non-tumorigenic mammary epithelial) were obtained from the American Type Culture Collection (ATCC, Rockville, MD, USA), and the human dermal fibroblasts (HDF) were purchased from ScienceCell Research Labs (San Diego, CA, USA). Most of the cells were grown in RPMI 1640 (BioWhittaker, Verviers, Belgium) with 10% FBS (Hyclone, Logan, Utah, USA), 1% Glutamax (Invitrogen, Carlsbad, CA, USA), and 1% Penicillin-streptomycin (BioWhittaker). Exceptions were DU145, HCT-116 and HDF which were grown in DMEM (BioWhittaker) with 10% FBS, 1% Glutamax, and 1% Penicillin-streptomycin, and MCF10A which was maintained in DMEM/F12 (BioWhittaker) containing 5% horse serum (Gibco Life Tech., Gaithersburg, MD, USA), 1 μg/ml insulin (Sigma, St Louis, MO, USA), 20 ng/ml EGF (BD Collaborative Research, Bedford, MD, USA), 1% Glutamax, and 1% Penicillin-streptomycin. The cells were passed approximately twice a week using trypsin/EDTA (BioWhittaker). In addition, the human keratinocyte cell line HaCaT [36] was cultured in Keratinocyte-SFM (Invitrogen Life Technologies) supplemented with 0.2 ng/ml human recombinant epidermal growth factor, 30 μg/ml bovine pituitary extract, and Penicillin-streptomycin. Further, whole cell lysates of human melanocytes (Mel) were kindly provided by Léon van Kempen (Dept. of Pathology, Radboud University Nijmegen Medical Centre, Nijmegen, The Netherlands).

### Transfections

Cells (A375 and MCC11) were seeded at 5 × 10^5 ^per well in a 6-well plate approximately 24 h prior to transfection. Transfections were performed using Lipofectamine 2000 (Invitrogen) following the manufacturer's protocol. Briefly, the transfection mixture contained a total of 4 μg plasmid (pTracer empty vector or pTracer containing a full-length human cystatin E/M cDNA (pCST6; kindly provided by Daniel Keppler, College of Pharmacy, Touro University-California, Vallejo, CA, USA)) and 10 μl Lipofectamine 2000 in 500 μl DMEM without FBS or antibiotics. This mixture was added to each well containing 2 ml DMEM with 10% FBS and no antibiotics. The following day, the transfection mixture was removed and the appropriate medium for experiments was added.

### Preparation of whole cell extracts for immunoblotting

Cells were washed 3 times with ice cold phosphate-buffered saline (PBS), scraped into PBS and pelleted by centrifugation at 2,500 × g for 5 min at 4°C. Pellets were resuspended in RIPA triple detergent lysis buffer (50 mM Tris (pH 8.0), 150 mM NaCl, 0.02% sodium azide, 0.1% SDS, 1% NP-40, 0.5% sodium deoxycholate, 1 mM PMSF, 2 μg/ml leupeptin, 2 μg/ml aprotinin, and 2 μg/ml pepstatin), and incubated on ice for 30 min. Lysates were centrifuged at 15,000 × g for 30 min. Supernatants were collected and protein concentration was determined using the BCA protein assay kit (Pierce, Rockford, IL, USA).

### Preparation of whole cell extracts for enzymatic activities

Cells were seeded at 5 × 10^5 ^per well in 6-well plates, and the following day the medium was replaced with 1 ml fresh medium without serum. After 24 h, adherent cells were washed 3 times in PBS before adding lysis buffer containing 100 mM sodium citrate, 1 mM disodium EDTA, 1% n-octyl-β-D-glucopyranoside, pH 5.8. Cell harvesting was assisted by a cell scraper and the collected lysates were frozen at -70°C. Following three cycles of freezing and thawing, the cell lysates were centrifuged at 10,000 × g for 5 min and the supernatants were applied to enzymatic activity analyses. Protein concentrations in the lysates were measured by the procedure described by Bradford [37], and performed according to the manufacturer (Bio-Rad Laboratories, Hercules, CA, USA) in a microplate reader measuring absorbance at 595 nm. Bovine serum albumin (Pierce) was used as a standard and all measurements were performed in triplicate.

### TCA precipitation of secreted proteins

The day following transfection or seeding in a 6-well plate, the medium was replaced with 1 ml fresh medium without serum. The conditioned medium was collected 48 h later and passed through a 0.45 μm filter. To precipitate secreted proteins, 0.5 ml 40% trichloroacetic acid (TCA) was added to 0.5 ml supernatant. Samples were vortexed thoroughly and incubated on ice for 1 h. Samples were spun at 14,000 × g for 30 min and supernatant was discarded. Pellets were re-suspended in ice-cold acetone using sonication and samples were incubated overnight at -20°C. Samples were washed twice by spinning at 14,000 × g for 5 min and re-suspension in acetone. Pellets were allowed to air dry for approximately 2 min and re-suspended in RIPA lysis buffer for SDS-PAGE.

### SDS-PAGE and immunoblotting

Whole cell extract (25 μg total protein), recombinant human legumain (5 ng; R&D Systems, MN, USA; 2199-CY) or pellets from TCA precipitation dissolved in RIPA lysis buffer were supplemented with Laemmli SDS-PAGE sample buffer (Invitrogen) and boiled at 100°C for 5 min. Samples were applied to 4-12% gradient gels in 2-(N-morpholino) ethanesulfonic acid (MES) buffer for SDS-PAGE (Invitrogen). Proteins were transferred to a 0.22 μm PVDF membrane (Millipore Corporation, Bedford, MA, USA) using the Xcell sure-lock mini-cell (Invitrogen) according to the manufacturers instructions. Following transfer, the membrane was blocked by incubation for one hour at room temperature in blocking buffer (TBS containing 5% non-fat dry milk and 0.1% Tween). Membranes were probed overnight at 4°C with primary antibody dissolved in blocking buffer at the following concentrations: cystatin E/M goat polyclonal (1:500; R&D Systems, AF1286), cystatin C goat polyclonal (1:1000; R&D Systems; AF1196), legumain goat polyclonal (1:1000; R&D Systems; AF2199), cathepsin B rabbit polyclonal (1:10,000; Calbiochem, San Diego, CA, USA), cathepsin L goat polyclonal (1:500; R&D Systems; AF952), α-tubulin mouse monoclonal (1:5000; Calbiochem) and β-actin mouse monoclonal (1:5000; Sigma). Membranes were washed twice for 5-10 min with washing buffer (TBS with 0.1% Tween). The membranes were subsequently probed with secondary antibody dissolved in blocking buffer at the following concentrations for one hour: horseradish peroxidase-conjugated goat anti-rabbit (1:5000), rabbit anti-goat (1:5000), or rabbit anti-mouse (1:5000) antibodies (DakoCytomation, Glostrup, Denmark). Subsequently, membranes were washed three times with washing buffer for 20 min and signals were developed with Super Signal West Dura Extended Duration Substrate (Pierce) according to the manufacturer's instructions, and visualized on either Medical X-ray films (Kodak, Rochester, NY) or the digital image station IS2000 (Kodak).

The immunobands visualized using a digital image station were quantified in the related software MI v.4.0.1 (Kodak), and those from X-ray films were scanned in a calibrated densitometer GS-800 (Bio-Rad) and quantified in the related software QuantityOne v.4.6.5 (Bio-Rad). All measured quantities were normalized using the corresponding loading control (α-tubulin) for each lane.

### ELISA

Serum-free conditioned media was collected after 48 h and passed through a 0.45 μm filter. The media was analyzed utilizing published ELISA procedures to determine the amount of cystatin C [38], cystatin E/M [39] and cystatin F [40] secreted by the cells. In addition, a specific ELISA was established for cystatin D using methods and materials described earlier for a mouse cystatin C ELISA [41]. Briefly, the IgG fraction of a high-titer polyclonal rabbit antiserum against recombinant human cystatin D [42] not showing cross-reactivity against cystatins A, B, C, D, E/M, F, S, SN and kininogen was used for construction of a double sandwich ELISA. Wells in polystyrene microplates (MaxiSorp; Nunc, Copenhagen, Denmark) were coated with the IgG fraction as catching antibody. Sample or isolated recombinant cystatin D [42] in dilutions from 0.2 to 100 ng/mL, was added to the wells. A portion of specific antibodies, affinity purified from the same IgG fraction of the anti-serum was biotinylated [43] and used as detecting antibody. Bound cystatin D was quantified using a streptavidin-horseradish peroxidase conjugate (Amersham Biosciences, Buckinghamshire, UK) and the color reaction with 2,2-azino-di-(3-ethyl-benzothiazolin-sulfonate) (Sigma) to detect peroxidase activity as absorbance at 405 nm.

### Matrigel invasion assay

Cells were seeded at 2 × 10^5 ^per well in 6-well plates and transfected with pCST6 or empty vector (pTracer) as described above. The following day, the transfection medium was removed and cells were grown in fresh RPMI (with 10% FBS) for approximately 6 hours. This medium was removed and cells were grown in RPMI (10% FBS) supplemented with [methyl-^3^H] thymidine (Amersham) for 24 h. Cells were detached with trypsin and 5 × 10^4 ^[^3^H]-labeled cells in 100 μl medium were added to each well in a 24-transwell permeable plate (8.0 μm polycarbonate membrane, 6.5 mm insert; Corning Incorporated, Corning, NY, USA) coated with Matrigel basement membrane matrix (1:60 dilution; BD Biosciences, San Jose, CA, USA). For migration studies the wells were left uncoated. The bottom wells received 600 μl medium with the same concentration of serum as the top well, either 1% or 10% FBS. Cells were removed from the upper and lower sides of the filter after 72 h incubation and [^3^H]-thymidine was counted in a liquid scintillation analyzer (Wallac Oy, Turku, Finland). The ratio of the counts obtained from the bottom versus total counts from both sides was used to determine migration and invasiveness.

### Measurements of proteolytic activities

Legumain activity was measured by cleavage of the substrate Z-Ala-Ala-Asn-NHMec (Department of Biochemistry, University of Cambridge, UK) as previously described [44,45]. Cell lysate (20 μl) was added to black 96-well microplates (No. 3915; Costar, Corning, NY, USA). After the addition of 100 μl buffer and 50 μl substrate solution (final concentration 10 μM), a kinetic measurement based on increase in fluorescence over 10 min was performed. Temperature was kept at 30°C and all measurements were performed in triplicate. Cathepsin B activity was measured in a similar manner except for the use of the substrate Z-Arg-Arg-NHMec (20 μM; Bachem AG, Bubendorf, Switzerland) and a buffer system described elsewhere [46,47]. Cathepsin L activity was measured using the substrate Z-Phe-Arg-NHMec (40 μM; Bachem AG) in the presence of the cathepsin B-specific inhibitor CA074 (0.25 μM; Sigma) as previously reported [46], with and without the addition of the cathepsin L-specific inhibitor Z-Phe-Tyr(t-Bu)-diazomethylketone (2.5 μM; Calbiochem).

Inhibitory activity towards legumain was measured in the culture media from all the cell lines and from the pCST6-transfected cells (A375 and MCC11). Inhibitory activity was evaluated by measuring the residual enzyme activity in a preparation of legumain after the addition of conditioned media. As a source of legumain, homogenized rat kidney was applied to a cation exchange column (Resource S, 1 ml, Amersham Biosciences) and eluted with a gradient of sodium chloride as described previously [48]. Peak legumain activity eluted at about 0.3 M NaCl and this partially purified legumain fraction was used as an enzyme source together with the substrate Z-Ala-Ala-Asn-NHMec. Legumain (20 μl) and media samples (17 μl) were added 83 μl buffer and incubated for 10 min, before 50 μl substrate solution was added (10 μM final concentration). Activity of the legumain preparation alone was used as positive control and the residual legumain activity after addition of conditioned media was measured and degree of inhibition calculated for each cell media.

### Statistical analysis

Data are shown as the mean ± standard error of the mean (SEM). Statistical significance of the Matrigel invasion assays was evaluated using the two-tailed Student's *t*-test, whereas two-way ANOVA was used to evaluate protease activities.

## Results

### Cystatin E/M expression in malignant melanoma cell lines

**Figure 1:**
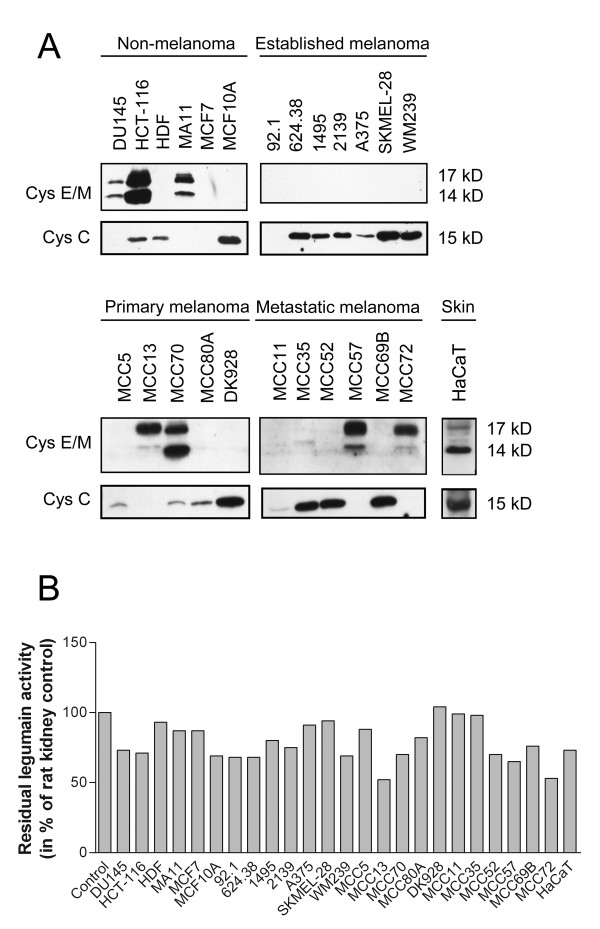
**Cystatin E/M and C secretions from various cell lines**: Common laboratory non-melanoma and established laboratory melanoma cell lines (top), and primary and metastatic melanoma cell lines established from patients, as well as skin control (bottom). (A) Equal amounts of serum free media were collected from 5 × 10^5 ^cells 48 h after changing from ordinary growth media and secreted proteins were concentrated by TCA-precipitation and subjected to SDS-PAGE and immunoblotting. The filters were stained with a cystatin E/M-specific (upper panels) or a cystatin C-specific (lower panel) antibody, respectively. (B) Inhibitory activity against legumain was measured in the conditioned media as residual legumain activity. A partially purified legumain fraction from rat kidney was mixed with conditioned media and the ability to cleave the substrate Z-Ala-Ala-Asn-NHMec was measured by fluorometry. Control bar (100%) represents activity in the rat legumain fraction without addition of conditioned media.

Although it is known that cystatin E/M is secreted by keratinocytes and is present in the skin [49], little is known concerning its expression in melanocytes or melanoma cells. To study this, we analyzed a panel of low passage cell lines derived from primary and metastatic lesions in addition to well-established melanoma cell lines. Conditioned media from an equal number of cells was collected 48 h after changing to serum-free media. Proteins in the supernatants were TCA-precipitated and examined for cystatin E/M and C expressions using immunoblotting. Several non-melanoma controls secreted cystatin E/M, including DU145, HCT-116 and MA11, and nearly equal amounts of unglycosylated (14 kD) and glycosylated (17 kD) forms were found (Fig. 1A, upper panels). Notably, in control cells derived from the skin, no cystatin E/M was secreted from human dermal fibroblasts (HDF), whereas HaCaT keratinocytes predominately secreted the non-glycosylated 14 kD cystatin E/M form. In contrast, the protease inhibitor was undetectable in media from all established melanoma cell lines examined, whereas strong immunobands were detected in two of the five primary melanoma lines (MCC13 and MCC70), and in two of the six lines derived from patients with metastatic disease (MCC57 and MCC72). Among the four melanoma lines secreting cystatin E/M, the glycosylated form (17 kD) was predominant compared to the non-glycosylated form (14 kD) in media from all but one cell line (MCC70).

Another cysteine protease inhibitor, cystatin C, was detected as one band (15 kD) in media from most of the cell lines examined (Fig. 1A, lower panels). However, in the melanoma lines where cystatin E/M was secreted, cystatin C was generally absent (MCC13, MCC57 and MCC72) or expressed at a very low level (MCC70). None of the cystatins were observed in the primary uveal melanoma cell line 92.1.

ELISA was used to quantify cystatin E/M and C in the conditioned media (Table 1). Among all the melanoma cell lines studied, cystatin E/M was only detected in the primary line MCC70 (0.28 ng/ml), whereas several of the non-melanoma lines displayed detectable levels (0.05-0.55 ng/ml; Table 1). Linear regression analyses showed a positive correlation between cystatin E/M levels measured by ELISA versus both the non-glycosylated (p < 0.0001) and the glycosylated (p = 0.0014) form of cystatin E/M on immunoblots after band intensities were scored 0-3 (exact quantification was difficult due to lack of representative loading controls for media samples). In addition, cystatin C was detected in media from all the cell lines analyzed except MCC13 (Table 1), with a positive correlation (p < 0.0001) between cystatin C as measured by ELISA and intensities of the immunobands (scored 0-3). Of other cystatins, cystatin F was only detected in media from MCC35 cells (5.6 ng/ml), whereas no detectable levels of cystatin D were observed in media from any of the cell lines.

Furthermore, the cellular levels of both cystatin E/M and C have been investigated by immunoblotting of cell lysates from all the cell lines included. No immunobands were obtained indicating that the cellular levels of both cystatins were below the level of detection (data not shown).

**Table 1:** Secretion of cystatin E/M and C into cell media measured by ELISA

Cell line	Cystatin E/M (ng/ml)	Cystatin C (ng/ml)
*Non-melanoma*		

DU145	0.11	2.7

HCT-116	0.55	8.0

HDF	n.d.^1^	7.4

MA11	0.33	2.0

MCF7	n.d.	10.2

MCF10A	0.05	11.2

*Established melanoma*		

92.1	n.d.	6.0

624.38	n.d.	10.1

1495	n.d.	7.0

2139	n.d.	7.5

A375	n.d.	8.8

SK-MEL-28	n.d.	9.6

WM239	n.d.	21.4

*Primary melanoma*		

MCC5	n.d.	6.3

MCC13	n.d.	n.d.

MCC70	0.28	6.3

MCC80A	n.d.	6.6

DK928	n.d.	9.9

*Metastatic melanoma*		

MCC11	n.d.	7.5

MCC35	n.d.	18.8

MCC52	n.d.	18.4

MCC57	n.d.	1.9

MCC69b	n.d.	13.3

MCC72	n.d.	1.5

^1^n.d.: not detectable

### Inhibition of legumain by conditioned melanoma media

The conditioned media from all cell lines was analyzed by fluorometry for their ability to inhibit the activity of a partially purified legumain fraction from rat kidney (Fig. 1B). Legumain inhibition was measured as residual legumain activity after addition of conditioned media to a fixed amount of purified legumain. Legumain activity in the enzyme preparation without addition of media was used as control (100% activity). Of the melanoma lines, MCC13, MCC57 and MCC72 showed the lowest residual legumain activity and thus highest inhibitory capacity. Cystatin E/M levels as detected by immunoblotting were scored (0-3) and a correlation analyzes performed, showing a statistically significant positive correlation in the whole cell panel (p = 0.0012) when comparing the level of cystatin E/M and the ability to inhibit rat legumain by the same cell media sample. In addition, inhibition of papain activity was measured similarly by cleavage of the substrate Z-Phe-Arg-NHMec with or without adding culture media from all cell lines studied (data not shown). Linear regression analyses showed that inhibition of papain correlated statistically significant with the cystatin C levels measured by ELISA (p = 0.0256), but not with the level of cystatin E/M.

### Cysteine protease expressions and activities in melanoma

**Figure 2:**
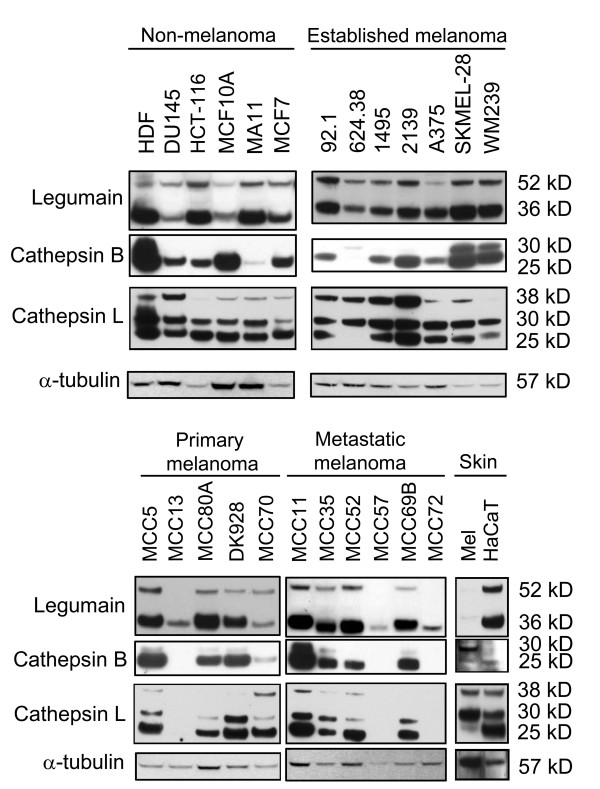
**Legumain, cathepsin B and L expressions in various cell lines**: Common laboratory non-melanoma and established laboratory melanoma cell lines (top), and primary and metastatic melanoma cell lines established from patients, as well as skin controls (bottom). Whole cell extracts were collected 24 h after seeding and equal amounts of total protein (25 μg) were used for SDS-PAGE and immunoblotting. The filters were stained with a legumain-specific (upper panels), cathepsin B- and L-specific (middle panels) or α-tubulin specific (lower panels) antibody, respectively.

Although the activities of cathepsin B and L in melanoma and other tumor types are well documented, the expression and activity of legumain in melanoma is relatively unknown. To determine expression of legumain, cathepsin B and L, whole cell lysates were prepared from the same panel of cell lines as used for cystatin E/M analysis (Fig. 1) following growth in ordinary media for 24 h. Immunoblotting was performed and two molecular bands for legumain were observed in most of the cell lines examined (Fig. 2, upper panels). The highest molecular weight form migrated at approximately 52 kD and represents prolegumain, while the smaller band of approximately 36 kD represents the active form. Three cell lines had only weak expression of the 36 kD band (MCC13, MCC57 and MCC72). Interestingly, these were the same cell lines that predominantly secreted the glycosylated form of cystatin E/M (17 kD) and showed the highest inhibitory activity against exogenously added legumain (Fig. 1).

Both cathepsin B and L were detected in most of the cell lines, except those predominantly secreting the glycosylated form of cystatin E/M (MCC13, MCC57 and MCC72; Fig. 2, middle panels). For cathepsin B, most of the positive melanoma lines expressed the 25 kD molecular weight form, whereas a few cell lines also showed lower levels of the 30 kD form. One cell line (624.38) expressed only low levels of the 30 kD form. Cathepsin L was generally expressed as three forms with molecular weights of 38, 30 and 25 kD, respectively. Interestingly, those melanoma cell lines from patients showing a strong expression of the 36 kD active legumain form, seemed to have a stronger expression of the 25 kD active cathepsin L form compared to the 30 kD form, although not statistically significant. This relationship also seemed to be expressed in HaCaT keratinocytes but not in the melanocyte (Mel) control (Fig. 2).

**Figure 3:**
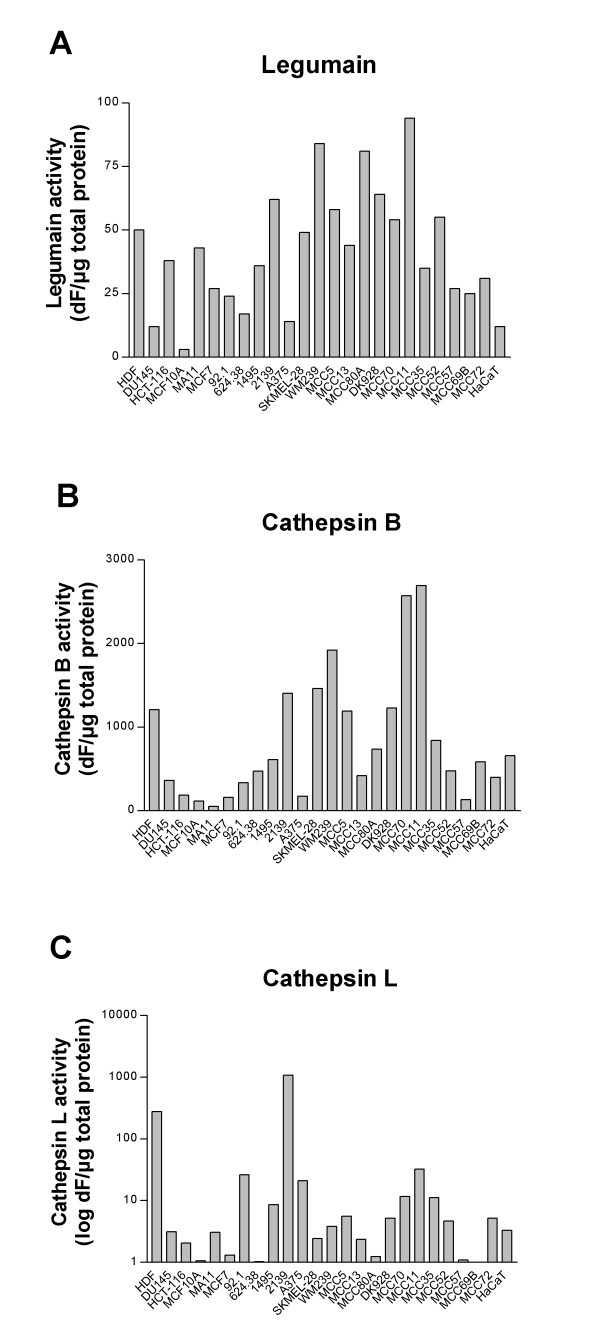
**Enzyme activities of legumain (A), cathepsin B (B) and cathepsin L (C) in various cell lines**. Whole cell extracts from common laboratory non-melanoma and established laboratory melanoma cell lines, primary and metastatic melanoma cell lines established from patients, and skin cell controls were collected 24 h after seeding. Legumain (A), cathepsin B (B) and L (C) activities in each cell lysate were measured by cleavage of peptide substrates and fluorometry (see Materials and methods for details). Note: The Y-axis in A and B are shown as dF/μg total protein whereas y-axis in C is shown as log dF/μg total protein.

In addition, legumain, cathepsin B and L activities in the cell lysates were measured by their ability to cleave peptide substrates (Fig. 3). Linear regression analysis of the whole group of cell lines revealed a positive correlation between legumain activity (Fig. 3A) and expression of the putative active 36 kD form (Fig. 2) and between cathepsin B activity (Fig. 3B) and expression of the 25 kD form (Fig. 2) (p = 0.0141 and 0.0351, respectively), as quantified using densitometric scanning of the immunoblots. In contrast, there were no statistical correlation between immunobands (Fig. 2) and activity (Fig. 3C) of cathepsin L. Two cell lines (HDF and 2139) showed particularly high cathepsin L activity (Fig. 3C), also reflected in strong immunobands (Fig. 2). Furthermore, legumain activity was analyzed in conditioned cell media but no active secreted legumain was detected.

### Over-expression of cystatin E/M suppressed legumain activity in melanoma

**Figure 4:**
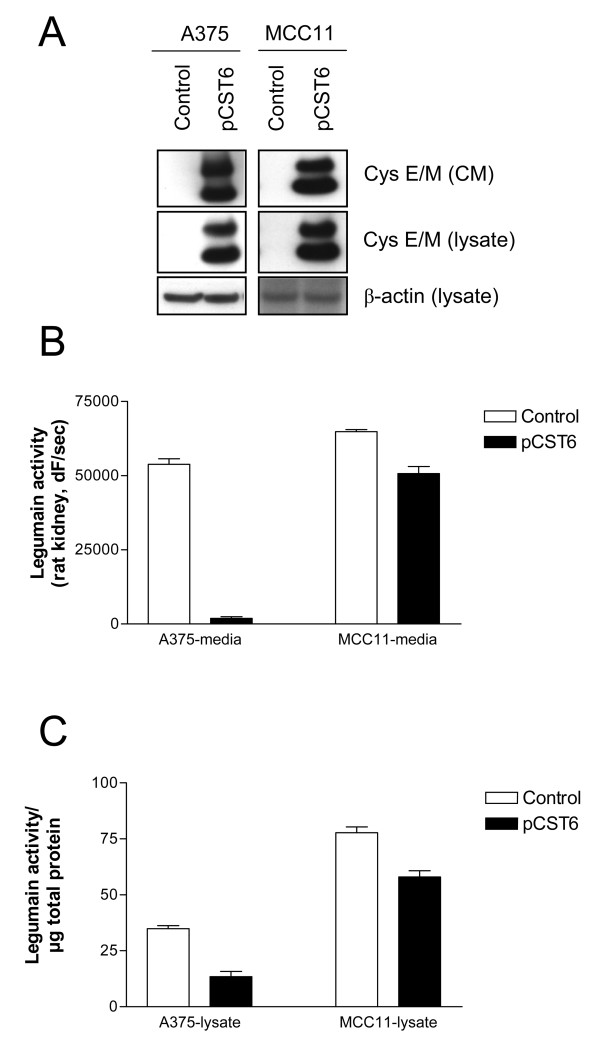
**Cystatin E/M over-expression in melanoma cells suppressed legumain activity**: A375 and MCC11 cells were transfected with pCST6 (closed bars) or empty pTracer vector (control; open bars), media were changed the following day, and serum-free conditioned media and cell lysates were collected 24 h later. (A) Immunoblots of cystatin E/M in conditioned media (CM) and lysates. Secreted proteins were TCA-precipitated prior to SDS-PAGE and immunoblotting. The filters were stained with a cystatin E/M-specific (upper and middle panels) or a β-actin specific (lower panel; loading control) antibody, respectively. (B) Inhibitory activity against legumain was measured in the conditioned media as residual legumain activity using fluorometry, a fixed amount of partially purified legumain from rat kidney and the substrate Z-Ala-Ala-Asn-NHMec (A375, p < 0.0001; MCC11, p = 0.002). (C) Activity of legumain in cell lysates measured by cleavage of Z-Ala-Ala-Asn-NHMec (A375, p < 0.0001; MCC11, p = 0.0012). Error bars represent the standard error of the mean (SEM).

To further investigate the relationship between cystatin E/M and legumain, two cell lines with no detectable levels of secreted cystatin E/M (A375 and MCC11; Fig. 1A; Table 1) and low inhibitory activity against legumain in the culture media (shown as high residual legumain activity, Fig. 1B) were utilized. A375 is a commonly used melanoma cell line, with intermediate expression of the 36 kD form of legumain (Fig. 2). MCC11 is a low-passage melanoma line established from a patient's lymph node metastases showing a high level of the 36 kD form of legumain (Fig. 2). These cell lines were transfected with the cystatin E/M expression plasmid pCST6 and the following day the transfection media was changed to serum-free media. Conditioned media and cell lysates were collected 24 h thereafter, and cystatin E/M expression was analyzed by immunoblotting. Whereas no immuno-reactive bands were found in vector-transfected cells, substantial levels of both cystatin E/M forms (14 and 17 kD) were observed both in conditioned media (Fig. 4A, upper panels) and in lysates (Fig. 4A, middle panels) from pCST6-transfected cells. No active legumain was found in conditioned media. The conditioned media was also examined for the ability to inhibit legumain activity using the partially purified legumain fraction from rat kidney as enzyme source (Fig. 4B). Media from both pCST6-transfected cell lines (closed bars) significantly inhibited legumain activity compared to empty vector-transfected controls (open bars). Media from transfected A375 cells almost completely inhibited (97%; p < 0.0001) the activity of the rat legumain fraction, whereas media from transfected MCC11 inhibited the enzyme activity by 22% (p = 0.002).

Legumain activity in the corresponding cell lysates from the pCST6-transfections was also measured, and the activity in both A375 and MCC11 was significantly decreased compared to controls (p < 0.0001 and p = 0.0012, respectively; Fig. 4C). A375 cells had a lower basal level of legumain activity than MCC11, which is reflected in the empty vector-transfected cells (control; Fig. 4C, open bars). Over-expression of cystatin E/M decreased the intracellular legumain activity by 62% and 25% in A375 and MCC11, respectively (closed bars). For comparison, there were no differences between the activities of cathepsin B in the same lysates when comparing control versus pCST6-transfected cells (not shown). Further, immunoblotting and densitometric scanning of the 36 kD legumain band in lysates from pCST6-transfected cells reflected the down-regulation of enzyme activity (not shown).

### Over-expression of cystatin E/M decreased invasiveness of metastatic melanoma

**Figure 5:**
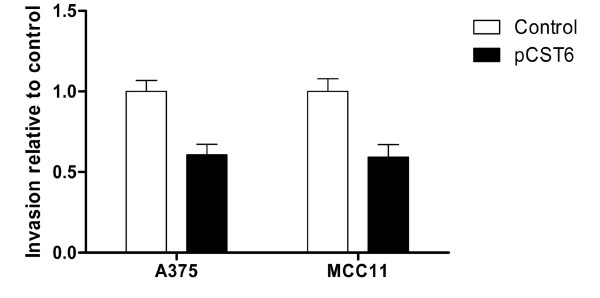
**Over-expression of cystatin E/M suppressed melanoma cell invasion**. A375 and MCC11 cells were transfected with pCST6 (closed bars) or empty vector (control; open bars) and migration through Matrigel coated transwell membranes was determined. The bars represent the average of four or three experiments for A375 and MCC11, respectively, with six replicate wells for each transfection group (A375, p < 0.01; MCC11, p < 0.05). Error bars represent the standard error of the mean (SEM).

Although it has been reported that cystatin E/M is capable of suppressing invasion of breast cancer [12,13], its ability to inhibit melanoma progression has not been investigated. To determine the contribution of cystatin E/M to the suppression of invasion, the pCST6-transfected cell lines A375 and MCC11 were examined for their ability to migrate through a Matrigel-coated filter of a transwell plate. Invasion was significantly decreased by 39% in A375 (p < 0.01) and 41% (p < 0.05) in MCC11 following transfection with pCST6 (Fig. 5). In contrast, there was no change in the rate of growth or motility when transwell plates without Matrigel were utilized (data not shown).

## Discussion

In this study, we provide data indicating that cystatin E/M suppresses invasiveness of melanoma cells, likely via the inhibition of the cysteine protease legumain known to be present in metastatic lesions. We have observed expression of cystatin E/M, legumain, cathepsin B and L in multiple melanoma cell lines, and an inverse correlation between secreted levels of the cysteine protease inhibitor and cellular activities of legumain and cathepsin B (but not cathepsin L) was detected. When cells lacking cystatin E/M were transfected with an expression vector containing cystatin E/M cDNA (pCST6), a significant decrease in cellular legumain activity was observed, and conditioned media collected from the transfected cells had high inhibitory activity against exogenously added legumain. Furthermore, following transfection with pCST6, invasiveness was significantly decreased as measured in a Matrigel invasion assay. Collectively, our data strongly suggests that cystatin E/M inhibits melanoma progression by suppressing legumain activity.

In a previous study comparing the gene expression profiles of primary and metastatic melanoma, it was shown that *CST6 *gene expression progressively decreases during disease progression [31]. When melanomas with intermediate Breslow's tumor thickness were compared to thick primary lesions, a substantial (nearly 70-fold) decrease in *CST6 *gene expression was observed. Furthermore, there was a significant decrease in *CST6 *expression between primary and metastatic melanoma when analyzed by microarray and quantitative RT-PCR. These data were the first to suggest that expression of *CST6 *decreases during progression to metastatic melanoma, a finding that is in agreement with previous studies in breast cancer [5].

We have identified several cell lines derived from both primary and metastatic melanoma that secrete cystatin E/M. However, despite data suggesting that cystatin E/M has tumor suppressive capabilities, we did not detect lower levels in the metastatic versus the primary cell lines. This was probably due to the relatively small number of cell lines at our disposal originating from primary tumors, and furthermore, that most of these lines were derived from rather large lesions possibly having a phenotype more closely resembling metastatic rather than primary melanoma. Interestingly, the glycosylated 17 kD cystatin E/M form predominated in the melanoma samples, whereas the unglycosylated 14 kD form predominated in HaCaT keratinocytes. To confirm that *CST6 *functions as a tumor suppressor gene in melanoma, further studies are needed to determine the extent of cystatin E/M expression and the glycosylation status in cultured melanocytes and thin primary tumors.

Although the expression of cystatin E/M in melanocytes has not been demonstrated, it has previously been shown that the protein is secreted from keratinocytes and thus present within the tumor microenvironment of the primary melanoma [49]. Herein, we show that cystatin E/M is secreted by HaCaT keratinocytes, whereas no cystatin E/M was observed in lysates of either melanocytes or HaCaT cells. In normal human skin, cystatin E/M has been observed to be constitutively expressed in the stratum granulosum, sweat glands, sebaceous glands and hair follicles [49,50]. During inflammation, its expression is detectable within several layers of the skin and into the stratum spinosum. Interestingly, basal cell and squamous cell carcinomas were found to express cystatin E/M only in well differentiated cells. However, there has been no histological examination of cystatin E/M expression in nevi, melanoma *in situ *or primary melanoma.

By immunoblotting, we detected cystatin E/M secretion from four melanoma cell lines (MCC13, 57, 70 and 72), and the conditioned media from these cells demonstrated the highest inhibitory activities against legumain. Surprisingly, we detected cystatin E/M by ELISA in media from only one of these melanoma cell lines. There are several potential explanations for this discrepancy, but the most likely being that there are fundamental differences between the applied methods. Immunoblotting measures denatured proteins while the ELISA attempts to detect the native cystatin E/M, possibly in complex with the interacting proteases. It is also possible that the amount of cystatin E/M in the media collected from the melanoma lines is below the limit of detection in the ELISA assay, as some cell lines showing strong expression on immunoblotting (e.g. HCT-116, MA11) had low amounts detected by ELISA. Furthermore, we utilized different antibodies in the two assays in this study and differences in the antibody affinity for the various forms of cystatin E/M are probable. The polyclonal antiserum used in the ELISA has, however, been demonstrated to recognize both the glycosylated and non-glycosylated forms of cystatin E/M (data not shown), making this explanation less likely. Nevertheless, there was a statistical positive correlation between the ELISA results for cystatin E/M and both molecular forms observed by immunoblotting.

Some interesting relationships were observed between the different expression profiles of cystatin E/M, cystatin C, legumain, cathepsin B and L. First, in most melanoma cell lines examined, we identified an inverse correlation between secreted cystatin E/M and C. The only exception was the ocular melanoma cell line 92.1, in which neither of the cystatin isoforms was detected. Likewise, a similar inverse correlation between secreted cystatin E/M and both pro- and active forms of the examined proteases in lysates was observed. Legumain was expressed at very low levels in cell lines secreting cystatin E/M (MCC13, MCC70, MCC57 and MCC72), while immunobands representing active legumain were high in all cells lacking the protease inhibitor. Similarly, with one exception (MCC70), there was no detectable expression of neither cathepsin B or L in the cells secreting cystatin E/M. Interestingly, this pattern was observed only in the melanomas and not in the cell lines derived from other cancer types. Although the expression levels of cystatin E/M and the proteases inversely correlated, an alternative explanation could be that cystatin E/M and protease expression levels are counter-regulated by some unknown mechanism. If so, the lower metastatic potential of the cystatin E/M expressing cells might be due to lower total expression levels of legumain, cathepsin B and L, respectively, and should be investigated further.

Although legumain has been shown to impart metastatic properties in several different types of cancer, the present study is the first to address the expression and activity of the protease in melanoma. In general, high legumain expression and activity was revealed in the examined panel of melanoma cell lines. Two immunobands were repeatedly observed, one migrating at approximately 52 kD corresponding to the inactive proform of the enzyme, and the other at approximately 36 kD corresponding to the active form [51]. The latter form was observed in the majority of the melanoma cell lines, and the intensity of this band correlated significantly with legumain activity in each cell line. Moreover, legumain has been shown to be involved in processing and activation of proteases such as cathepsin L [52]. In our study, comparisons of band intensities of legumain and cathepsin L did not statistically correlate, but the melanoma cell lines derived from patient biopsies seemed to express a stronger 25 kD mature cathepsin L immunoband in the cell lines with a strong 36 kD active legumain band.

To determine the influence of cystatin E/M on protease activity and invasion, two cell lines (A375 and MCC11) with no basal secretion of cystatin E/M were transfected with pCST6. Cystatin E/M over-expression resulted in significantly increased ability to inhibit exogenously added legumain by the conditioned media, as well as decreased cellular legumain activity. The percentage decrease in legumain activity was greater in lysates from A375 than MCC11, possibly due to the lower basal level of legumain activity in A375. Surprisingly, cathepsin B was not affected by over-expression of cystatin E/M in these cells. Furthermore, a significant inhibition of invasive capacity was observed in both cell lines following transfection with pCST6. Since enhanced expression of cystatin E/M did not influence on the cell ability to migrate, the observed effect on invasion is most likely due to cystatin E/M-mediated inhibition of legumain activity. However, it is possible that the decreased invasiveness could in part result from effects on proteases other than legumain.

Although the majority of reports on cystatin E/M have indicated that it suppresses tumor growth and metastasis, there have been some conflicting data regarding the role of the protease inhibitor in cancer. It has been reported that cystatin E/M is up-regulated during the progression from primary to metastatic oropharyngeal squamous cell carcinoma [53]. In addition, silencing of cystatin E/M markedly increased proliferation rate, *in vitro *motility and Matrigel invasiveness [54]. A study in pancreatic cancer showed that knockdown of cystatin E/M with siRNA suppressed *in vitro *growth whereas over-expression increased growth rate and tumor burden *in vivo *in pancreatic cancer [55]. The authors proposed that the growth enhancing properties of cystatin E/M result from inhibition of cathepsin B facilitated apoptosis. However, we could not detect any changes in cathepsin B activity when over-expressing cystatin E/M in melanoma cells. Despite these reports, the majority of studies in other tumor types have found that cystatin E/M exerts a suppressive effect on carcinogenesis and demonstrated growth and metastasis suppressing properties in both breast cancer and glioma [12-14].

## Conclusions

In summary, we have shown that cystatin E/M acts as a regulator of proteolytic activity of legumain and suppressor of invasion in metastatic melanoma. Further studies are needed to elucidate the role of both cystatin E/M and legumain in melanoma, including their expressions during different stages of the disease and their roles in tumor formation and metastasis *in vivo*. The data presented provide new insight into the role of cystatin E/M in protease regulation and invasion in malignant melanoma.

## List of abbreviations used

Cys: cystatin; MCC: Moffitt Cancer Centre; pCST6: cystatin E/M (CST6)-encoding plasmid

## Competing interests

The authors declare that they have no competing interests.

## Authors' contributions

JJB carried out cell culturing and harvesting, immunoblotting of cystatins, transfections, Matrigel invasion assays and statistical analysis, and participated in design of the study. MHH carried out immunoblotting and densitometry scanning of cystatins and proteases, participated in the statistical analysis and performed the final design of the figures. HTJ performed chromatography of rat legumain, participated in protease activity measurements and study design. AIR established several of the used melanoma cell lines and participated in the original conception and design of the study. MA performed ELISA measurements. ØF participated in the original design of the study. GMM participated in design and progression of the study. RS participated in protease activity measurements and statistical analysis, as well as in design and coordination of the study. JJB, GMM and RS drafted the manuscript, whereas all authors have contributed to, read and approved the final manuscript.

## Pre-publication history

The pre-publication history for this paper can be accessed here:

http://www.biomedcentral.com/1471-2407/10/17/prepub
